# Influence of Epoxy Functional Chain-Extenders on the Thermal and Rheological Properties of Bio-Based Polyamide 10.10

**DOI:** 10.3390/polym15173571

**Published:** 2023-08-28

**Authors:** Rafael Erdmann, Mirko Rennert, Thomas Meins

**Affiliations:** Institute for Circular Economy of Bio:Polymers at Hof University (ibp), Hof University of Applied Sciences, Alfons-Goppel-Platz 1, 95028 Hof, Germany; mirko.rennert@hof-university.de (M.R.); thomas.meins@hof-university.de (T.M.)

**Keywords:** polyamide 10.10, chain-extender, Joncryl^®^, epoxy functionalization, reactive modification, branched polymer

## Abstract

Bio-based polyamide 10.10 (PA 10.10) has excellent properties compared to other bio-based polymers such as polylactic acid (PLA) or polyhydroxyalkanoates (PHAs) and is therefore used in more technical applications where higher strength is required. For foam and filament extrusion, a good balance between strength and stiffness of the polymer is needed. Therefore, two commercial chain-extenders (Joncryl^®^ ADR types) with different epoxy functionalities are used to modify the melt properties of PA 10.10. The chain-extenders are used in a concentration range up to 1.25 wt.%. The range of glass transition temperature widens with increasing Joncryl^®^ content, and the apparent activation energy shows a maximum at a concentration of 0.5 wt.%. Furthermore, the melting temperatures are constant and the crystallinity decreases with increasing chain-extender content due to the formation of branches. During the second heating run, a bimodal melting peak appeared, consisting of α-triclinic and pseudo γ-hexagonal crystals. The weight average molar masses (*M_w_*) measured by gel permeation chromatography (GPC) increased linearly with increasing ADR 4400 content. In contrast, the compounds containing ADR 4468 show a maximum at 0.5 wt.% and it begins to decrease thereafter. The rheological data show an increase in viscosity with increasing chain-extender content due to branch formation. ATR spectra of the compounds show a decrease at the wavelength of the primary (3301 cm^−1^) and secondary (1634 cm^−1^) (-NH stretching in PA 10.10) amine, indicating that chain-extension, e.g., branching, takes place during compounding.

## 1. Introduction

Polyamides are widely used thermoplastic polymers for engineering applications due to their excellent mechanical properties. Nowadays, various polyamides can be produced due to versatile bio-based feedstock and platform chemicals derived from them [1,2,3,4]. While a wide range of different polyamides is possible, not all of them are produced at a large commercial scale. Depending on the product and its properties, polyamides must be modified to reach the specific properties.

A suitable way to modify polymer properties, in general, is reactive modification during the compounding process [5,6,7,8], called reactive extrusion. The polymer most commonly studied for this modification process is poly (ethylene terephthalate) (PET). This is due to the large amount of material used for beverage bottles, which are kept in a closed recycling loop [9].

Polyamides are generally modified with bisoxazolines [10,11,12,13,14] and biscaprolactam [14,15,16], but other modifiers based on isocyanates [17] and epoxides [14,18] have also been reported. Polyamide 10.10 is fully bio-based and has comparable properties to petrochemical-based polyamide 6 (PA 6). It is typically used for filaments or foam applications, but is also frequently modified in advance [14,19]. In order to extend the application range of bio-based polyamide 10.10, it needs to be modified due to the lack of melt strength, which is crucial for this type of application. The first modification of polyamide 10.10 with epoxides by reactive extrusion was carried out by Qian et al. [20]. They used diepoxide 711 and diepoxide TDE85 as chain-extenders. An increase in torque and corresponding melt viscosity was observed for both chain-extenders. The reaction between the epoxides and the amides of PA 10.10 took place within 5 min. This means that the modification of PA 10.10 could be achieved by reactive extrusion. It was also found that the reaction with diepoxide TDE85 was faster than with diepoxide 711 due to an additional epoxide group.

In other publications dealing with the modification or blending of polyamide 10.10, epoxides are used as compatibilizers between PA 10.10 and bio-PET [21] or PLA [22], or as modifiers for PLA subsequently blended with polyamide 10.10 [23,24].

The aim of this study is to answer the question of to what extent the molar mass of PA 10.10 can be increased with commercially available chain-extenders (Joncryl^®^ types) and whether gel particles form in PA 10.10 when the recommended concentration range is used, and what rheological and thermal properties result. Further steps would be to analyze the melt strength of the modified PA 10.10 compared to pure PA 10.10 using rheotens tests and to investigate whether filaments can be formed from it. The final step would be to use the filaments as reinforcement fiber in the PA 10.10 matrix to obtain a self-reinforced plastic. To our knowledge, there is no research on this topic for PA 10.10. Cellulose [25,26], glass [27,28], basalt [29] and carbon fibers [30] are currently used to reinforce PA 10.10 to improve its properties.

## 2. Materials and Methods

### 2.1. Materials

**Figure 1:**
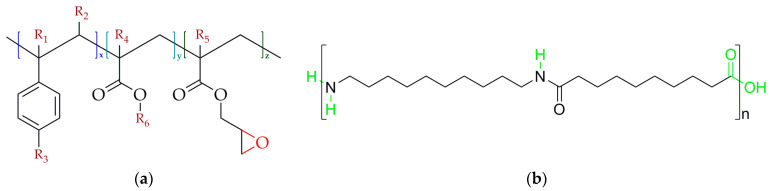

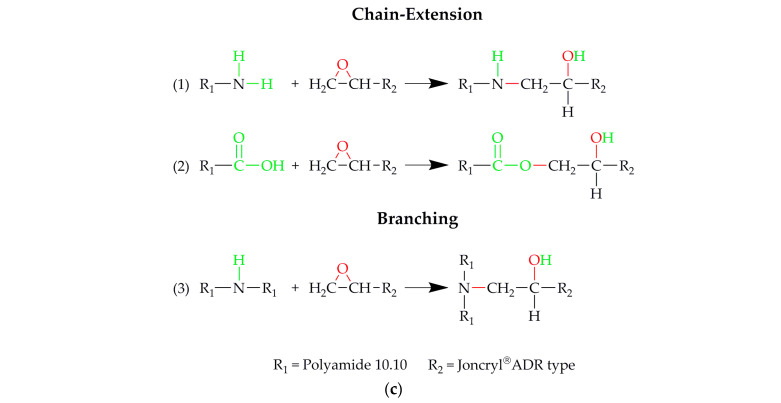
(**a**) General structure of the various epoxy-based Joncryl^®^ types (R_1_–R_5_ are H, -CH_3_, a higher alkyl group or combinations of those), R_6_ is an alkyl group (x, y and z are each between 1 and 20) [33]; (**b**) structure of polyamide 10.10 and (**c**) reaction mechanism between the epoxy functional group of Joncryl^®^ and the primary and secondary amines as well as the carboxylic group of the polyamide 10.10.

The used polyamide 10.10 (Vestamid^®^ Terra DS 16 natural) with a bio-based content of 100% and a molar mass (*M_n_*) of approximately 20 kg mol^−1^ was purchased from Evonik Operations GmbH, Essen, Germany. This polyamide 10.10 type shows a specific gravity of 1.05 g·cm^−3^. The supplier denotes the glass transition and melt temperature of polyamide 10.10 (DS 16) at 37 °C and 200 °C (dry condition). The maximum water absorption at room temperature is specified at 1.8% (Evonik test method). For chain-extension, e.g., branching of the PA 10.10, two different multifunctional Joncryl^®^ types, ADR 4400 and ADR 4468 from BASF SE, Ludwigshafen, Germany (distributed by BTC Europe GmbH, Monheim am Rhein, Germany), are used [31,32]. The general structure of Joncryl^®^ and polyamide 10.10 as well as their chain-extension and branching reaction are shown in Figure 1a–c.

Both Joncryl^®^ types (Table 1) are similar and only differ in their content of functional groups. Depending on the literature source, the weight average functionality $f_{w}^{CE}$ (ADR 4400 ~ 14; ADR 4468 ~ 24) [34] or the number average functionality $f_{n}^{CE}$ (ADR 4400 of 5 and ADR 4468 of 9) [33] are given, the latter of which has a lower value. Both Joncryl^®^ types show epoxy functions, which can react with hydroxyl (-OH) and more preferably with carboxyl (-COOH) groups. Epoxy groups can furthermore react with the primary (-NH_2_) and secondary amine (-NH) groups which are present in PA 10.10. Both Joncryl^®^ types react quickly and reach a conversion of 99% at 200 °C and 120 s or 280 °C and 30 s. According to the manufacturer’s recommendation [31,32], the average processing conditions were set at ~260 °C with a low screw speed (250 min^−1^) as well as low throughput (2.5 kg h^−1^), so the dwell time was at least 120 s, facilitating a full conversion of the Joncryl^®^ during processing.

Due to slight differences in molar mass and more importantly in functionality, weight concentration (wt.%) is not a suitable parameter for comparing the chain-extender quantitatively. Rather, the molar mass and functionality of the chain-extenders, as well as the corresponding quantities of PA 10.10, have to be taken into account. A suitable and therefore more reliable parameter to distinguish the effectiveness of the chain-extenders is their equivalent ratio Φ (Equation (1)).

(1) $$\Phi =\frac{f_{n}^{CE}/M^{CE}\cdot \varphi^{CE}}{f^{p}/M^{p}\cdot \varphi^{p}}\;$$

$f_{n}^{CE}$ and $f^{p}$ are the number average functionalities of the chain-extenders and PA 10.10, $M^{CE}$ and $M^{P}$ are the number average molar masses of the chain-extenders and PA 10.10 and $\varphi^{CE}$ and $\varphi^{p}$ are the weight concentrations of the corresponding chain-extender and PA 10.10.

### 2.2. Processing

#### 2.2.1. Compounding of Polyamide 10.10 including Different Chain-Extender Types

Polyamide 10.10 compounds with varying chain-extender content were prepared in a co-rotating intermeshing twin-screw extruder (Labtech Engineering Co., Ltd., Samutprakarn, Thailand), with a screw diameter of 20 mm and an L/D ratio of 44. The throughput was kept constant at 2.5 kg h^−1^, and the screw speed was set at 250 min^−1^. A special screw design with kneading and shear elements was used. Table 2 shows the temperature profile and main characteristics of the screw design.

Polyamide 10.10 as a hydrophilic biopolymer was dried for 4 h and 80 °C in a dry-air-dryer (LUXOR 50 from motan-colortronic GmbH, Friedrichsdorf, Germany) prior to processing. The chain-extender in its crystal form was ground and premixed with polyamide 10.10 granulate. The dry blend was then introduced through the main feeder. The melt was extruded through a dual-strand die with a diameter of d_o_ = 3 mm before cooling down in a water bath and pelletized afterwards.

#### 2.2.2. Heatpressing of Thin Films

For rheology measurements, thin films with thicknesses of ~500 µm must be produced. A press from Vogt Labormaschinen GmbH, Berlin, Germany, with heated upper and lower plates was used for this purpose. The modified compounds were dried beforehand as described in Section 2.2.1. Thirty grams of each mixture was weighed and placed between two metal slabs with PTFE sheets as an interlayer to ensure good release of the compressed films. The slabs were placed between the heated plates (220 °C on both plates) for 5 min to ensure heat transfer and complete melting of the compounds. After the melting process, the compound was compressed for 5 min at 20 bar (290 psi). The hot slabs were removed from the press and placed between water-cooled heat sinks to ensure continuous cooling of the ~400–600 µm thin films to ambient temperature. The films were removed from between the slabs, die-cut into 25 mm diameter discs and then placed in a desiccator to prevent water uptake.

### 2.3. Test Methods and Sample Preparation

#### 2.3.1. Differential Scanning Calorimetry (DSC)

To analyze the different phase transitions of the modified PA 10.10, differential scanning calorimetry (DSC) was performed with a DSC 214 Polyma^®^, NETZSCH-Gerätebau GmbH, Selb, Germany, which was equipped with an intracooler that allows measurements between −70 °C and 600 °C. Calibration of the device was carried out with the following material standards: indium, tin, bismuth and zinc at a heating rate of 20, 30, 40 and 50 K min^−1^. The compounds and the raw materials, which had an initial weight of 10 ± 0.5 mg, were placed in an aluminum pan with a pierced lid. The measurements were carried out under nitrogen atmosphere to avoid oxidation reactions and were performed at the same heating rates as the calibrations. The purge and protective gases were set at flow rates of 40 and 60 mL min^−1^ as recommended by NETZSCH-Gerätebau GmbH. The samples were cooled to −70 °C, heated up to 250 °C (with a heating rate of 20 K min^−1^) or 270 °C (at rates of 30–50 K min^−1^), cooled again to −70 °C and reheated to 250 °C or 270 °C. Between the heating and cooling periods, the samples were kept under isothermal conditions at the lowest and highest temperatures (for 5 min at −70 °C and 250 °C or 270 °C), respectively. The measurements were analyzed via the software NETZSCH Proteus^®^ 8.0.3, NETZSCH-Gerätebau GmbH, Selb, Germany.

The crystallinity $X_{c}$ of the bio-based PA 10.10 compounds was calculated according to the following Equation (2):

(2) $$X_{c}=\frac{\left({∆H}_{m}-{∆H}_{cc}\right)}{{∆H}_{m}^{0}}\;\cdot 100$$

${\Delta H}_{m}$ represents the melt enthalpy, ${\Delta H}_{cc}$ the enthalpy of the cold crystallization if it appears in a spectrum and ${∆H}_{m}^{0}\;\left(PA10.10\right)=244\;Jg^{-1}$ [3] is the enthalpy by 100% crystallinity of PA 10.10.

The apparent flow activation energy $E_{a}$ at the glass transition region can be assessed with the empirical Equation (3) of Moynihan et al. [35].

(3) $$\frac{-E_{a}}{R}=\frac{d\ln\beta }{d\left(1/T_{g}\right)}$$

$E_{a}$ represents the apparent flow activation energy at the glass transition temperature, R is the universal gas constant, ln⁡β are the heating rates and $1/T_{g}$ is the inverse of the measured glass transition temperatures.

A further approach to determine the apparent flow activation energy is given by Kissinger (Equation (4)):

(4) $$\frac{-E_{a}}{R}=\frac{d\ln\left(\beta /{T_{g}}^{2}\right)}{d\left(1/T_{g}\right)}$$

The notations of the Kissinger model are the same as those of Moynihan et al.’s.

#### 2.3.2. Size Exclusion Chromatography (SEC)

To determine the molar mass, an Agilent SEC 1100 measuring device from Agilent Technologies Inc., Santa Clara, CA, USA, was used. The device includes an isocratic pump (G1310A), an autosampler (G1313A), RI detector (1362A) and a column thermostat (G1316A). Additionally, a PL-DG-2 degassing unit from the company Polymer Laboratories Inc., Los Angeles, CA, USA, was implemented. The column unit consists of three PFG columns from the company PSS GmbH, Mainz, Germany, with a spacer material of modified silicon dioxide with porosities of 1000 Å, 300 Å and 100 Å and a PFG pre-column. All columns have the same cross-section of 0.78 × 30 cm^2^. As the eluent, hexafluoro-2-propanol (HFIP) with 50 mmol of natrium trifluoroacetate with a flow rate of 1 mL min^−1^ and measuring temperature of 30 °C was used. The calibration was carried out with PMMA standards within a molar mass range of 800 up to 2,200,000 g mol^−1^. Each sample (16 mg) was dissolved in 8 mL of the above-mentioned eluent for 12 h under constant vibration and filtrated afterwards with a PTFE filter (pore size 0.45 µm). The injected sample volume was 100 µL and was analyzed twice.

#### 2.3.3. Oscillation Rheology (Plate–Plate)

Rheometric measurements were performed to determine the viscoelastic behavior of the modified bio-based polyamide 10.10 compounds. An HR-20 rheometer with a plate–plate geometry of (d = 25 mm) from TA Instruments, Hüllhorst, Germany was used for all measurements. The compressed film discs described in Section 2.2.2 were used. To heat the samples above their melting temperature ~200 °C, a Smart Swap™ ETC, Hüllhorst, Germany, oven with a convection heating element was used. Prior to the main characterization (frequency sweeps), thermal stabilization tests (isothermal measurement over time) and amplitude measurements (to determine the non-linear region) were performed. To generate master curves and determine the apparent activation energy, isothermal measurements were performed at 220, 230, 240 and 250 °C with a strain amplitude of 1%, an overall soak time of 360 s and frequencies between 0.1 and 100 Hz (0.62–628 rad s^−1^) and analyzed with the software TRIOS Version 5.5.1.5 (TA Instruments, Hüllhorst, Germany).

To fit the viscosity data, the Carreau–Yasuda model was applied (Equation (5)):

(5) $$\eta =\eta_{0}\cdot {\left[1+{\left(\lambda \cdot \dot{y}\right)}^{a}\right]}^{\frac{n-1}{a}}$$

$\eta_{0}$ represents the zero shear viscosity, λ is a time constant, $\dot{\gamma }$ is the shear rate, n is the flow index (0 < n < 1) and a is a dimensionless factor and describes the transition between the first Newtonian plateau and the shear thinning behavior.

To create viscosity master curves, the temperature shift factor $a_{T}$ has to be assessed according to Equation (6):

(6) $$a_{T}=\frac{\eta_{0}\;(T)}{\eta_{0}(T_{ref.})}$$

$a_{T}$ is the temperature shift factor and $\eta_{0}$ the zero shear viscosities at the measuring temperature $\left(T\right)$ or reference temperature $\left(T_{ref}\right)$.

To estimate temperature dependency of the viscosity, the apparent flow activation energy can be determined according to the Arrhenius law (Equation (7)):

(7) $$\eta_{0}\;e.g.\;\;a_{T}=exp\cdot \left[\frac{E_{a}}{R}\cdot \left(\frac{1}{T}-\frac{1}{T_{ref}}\right)\right]$$

$a_{T}$ is the temperature shift factor, $E_{a}$ is the flow activation energy, R is the universal gas constant, T is the absolute temperature and $T_{ref}$ is the reference temperature.

#### 2.3.4. ATR-FTIR Spectroscopy

The attenuated total reflection (ATR) technique was applied to quantitatively analyze whether chain-extension, e.g., branching of polyamide 10.10, occurred during the compounding step. This technique can also provide information about the crystal form present (α-triclinic or pseudo γ-hexagonal) and, for example, its ratio in the PA 10.10 compound [3].

For ATR and FTIR measurements, a Bruker Tensor 27 with a SpectraTech solution ATR cell from Bruker Optics GmbH & Co. KG, Ettlingen, Germany was used. The AT spectra were analyzed with the software OPUS (Version 7.5.18), also from Bruker Optics GmbH & Co. KG, Ettlingen, Germany, in the wavenumber region between 400 and 4000 cm^−1^. For every sample, 3 measurements were conducted with 16 background and 64 sample scans with a resolution of 4 cm^−1^ (10 kHz). The Joncryl^®^ types were analyzed in transition mode (FTIR). For this purpose, 200 mg of potassium bromide (KBr) was weighed and mixed with ~10 mg of each Joncryl^®^ type which had been ground beforehand. The mixture was then pressed into a disc under vacuum at 10 bar (145 psi) for one minute using the SpectroPress™ from Chemplex Industries Inc., Palm City, FL, USA. The measurements were directly conducted afterward with the number of scans mentioned previously. Table 3 shows the characteristic wavenumbers of bio-based PA 10.10 as well as those of the two Joncryl^®^ types.

## 3. Results and Discussion

### 3.1. Differential Scanning Calorimetry (DSC)

**Figure 2:**
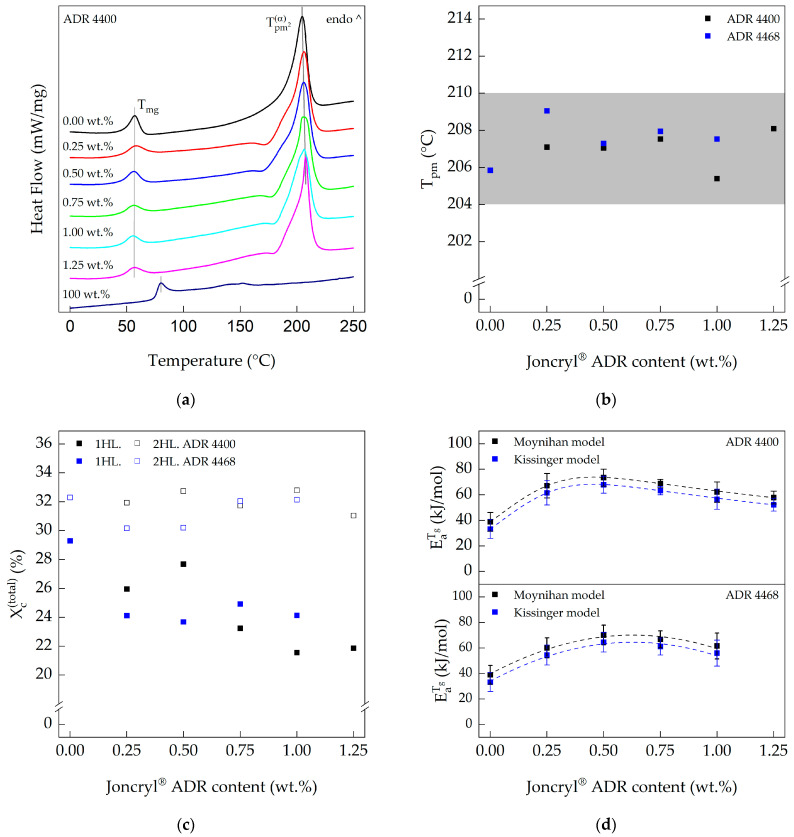
(**a**) DSC curves of Joncryl^®^ ADR 4400-modified PA 10.10 (1 heating rate; 20 K min^−1^); (**b**) melting temperatures of the PA 10.10 compounds as a function of Joncryl^®^ ADR modifier concentration (1 heating rate; 20 K min^−1^); (**c**) crystallinity of the modified PA 10.10 compounds (first and second heating run; heating rate 20 K min^−1^); (**d**) apparent flow activation energy of the Joncryl^®^-modified PA 10.10 compounds at glass transition region (1. heating run).

Figure 2a,b show the glass transition and melting temperatures of the neat PA 10.10 and various PA 10.10 Joncryl^®^ compounds. The results of the first heating rate were selected based on the intended application as filament for self-reinforced polyamide 10.10.

As the Joncryl^®^ content increases, the glass transition temperature range widens due to the increasing degree of branching. The peak of the melting temperature (Table 4) is not influenced by chain-extender content or the type of chain-extender used, but by the cumulative enthalpies of fusion, which decreased with the increasing degree of branching during the first heating run.

In the first heating run, the crystallinity (Figure 2c) of the compounds is influenced by the concentration of chain-extender. An increasing amount of chain-extender leads to increased branching and formation of free volume (amorphous regions) during the reactive extrusion step. After this step, the melt is rapidly cooled in a water bath and pelletized. Therefore, the melt does not have enough time to crystallize sufficiently.

**Figure A1:**
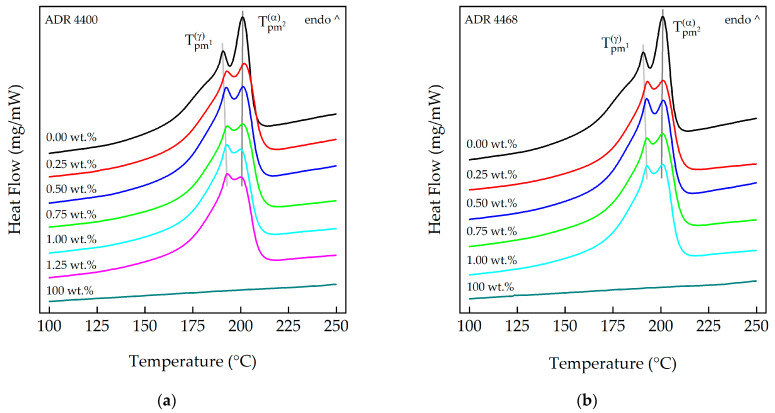
(**a**) DSC curves of Joncryl^®^ ADR 4400–modified PA 10.10 and (**b**) DSC curves of Joncryl^®^ ADR 4468–modified PA 10.10 (2. heating run; heating rate 20 K min^−1^).

In the second heating run, the melt has more time for crystallization, but this is also influenced by the type and content of the chain-extender or the degree of branching (Appendix A, Figure A1 and Table A1). With increasing chain-extender content, two crystal structures, pseudo γ-hexagonal and α-triclinic, are formed in polyamide 10.10 [47]. With increasing Joncryl^®^ content, the pseudo γ-hexagonal crystal form becomes more dominant, but the total enthalpy of fusion of the α- and γ-crystals remains almost constant (Table A1) [30].

The apparent flow activation energy in the glass transition region is shown in Figure 2d. The measured flow activation energy for PA 10.10 (processed) was 39 ± 7.4 kJ mol^−1^ (Moynihan model), which is in good agreement with the energy of 42 kJ mol^−1^ determined by Pagacz et al. [3]. Increasing the Joncryl^®^ content results in an increase in the apparent flow activation energy with a maximum of 73.6 ± 6.6 kJ mol^−1^ ADR 4400 and 70.1 ± 7.7 kJ mol^−1^ ADR 4468 at an approx. 0.5 wt.% chain-extender concentration, determined according to Equation (3).

### 3.2. Size Exclusion Chromatography (SEC)

**Figure 3:**
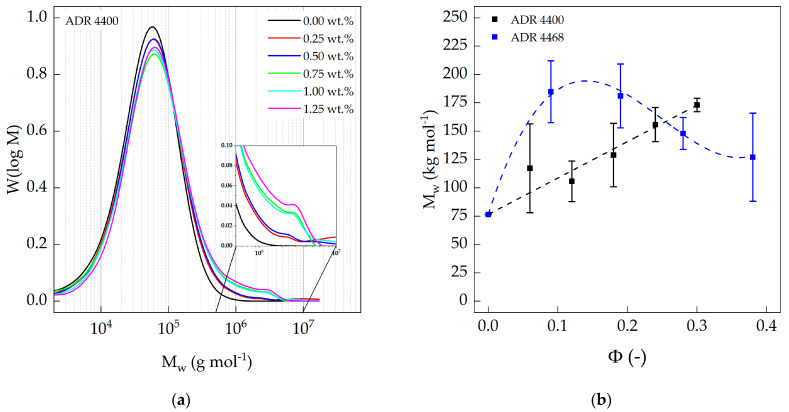
(**a**) Molecular weight distribution of ADR 4400-modified polyamide 10.10, as a function of the molar mass, determined via refractive index (RI detector); (**b**) weight average molar mass of the polyamide 10.10 compounds as a function of the equivalent ratio.

Figure 3a shows the molar mass distribution of PA 10.10 with increasing Joncryl^®^ ADR 4400 content. The average molar mass and molar mass distribution (D) increased with increasing chain-extender (Joncryl^®^ ADR 4400) content. It is known that an overdose of Joncryl^®^ leads to the formation of gel particles, depending on the combination of matrix material and Joncryl^®^ type as well as the equivalent ratio [48,49]. All modified PA 10.10 compounds were completely dissolved in HFIP prior to SEC analysis. Therefore, no gelation occurred during the compounding step. Moreover, the equivalent ratio Φ of PA 10.10 and ADR 4400 or ADR 4468 is relatively low at 0.30 and 0.38, respectively. This means that, statistically, the amount of amino groups (-NH) remaining in the polyamide is much higher compared to the reactive groups of the chain-extender. Furthermore, with increasing content of chain-extenders, a high molecular tail end is formed (Figure 3a). This was also observed by Härth et al. [50,51,52] for PET modified with Joncryl^®^. The same effects could be observed for epoxy-modified polymers such as PLA [53,54,55] and PA 6 [19].

The weight average molar masses of the modified PA 10.10 Table 5 determined by SEC (Figure 3b) can be directly correlated with the processing parameters such as torque and die pressure. Joncryl^®^ ADR 4400 has a lower concentration of functional groups compared to ADR 4468. During processing, torque [56], but more significantly die pressure, increased with increasing ADR 4400 content (Table A3) due to branching effects in the material, indicating an increase in weight average molar mass (Figure 3b). Joncryl^®^ ADR 4468 contains nine functional groups and thus almost twice as many as Joncryl^®^ ADR 4400. Therefore, a higher torque and die pressure were expected. Instead, at a content of 0.25 wt.% chain-extender, there is only a slight increase in torque [57] and die pressure, which then decreased again. This trend was also observed for the average molar mass (Figure 3b). Higher functionality of the chain-extender leads to higher shear forces due to increased branching and may in turn promote further chain degradation during processing [58]. The same effect was described by Cailloux et al. [54] for polylactic acid (PLA) compounds. A too-high concentration of Joncryl^®^ can also have a negative effect on the strain hardening behavior, as Standau et al. found for PBT [59].

**Table 5:** SEC results of the modified polyamide 10.10 compounds ^1^.

Compound	PA 10.10/ADR Ratio	Φ	$\bar{M_{w}}$	$\bar{M_{n}}$	D
	[wt.%]	[-]	[kg mol^−1^]	[kg mol^−1^]	[-]
PA 10.10 (processed)	100	0.00	77 ± 0.3	23 ± 0.9	3.3 ± 0.1
PA 10.10/ADR 4400	100/0.25	0.06	117 ± 39.2	27 ± 0.2	4.3 ± 1.4
	100/0.50	0.12	106 ± 17.8	27 ± 2.1	4.0 ± 0.4
	100/0.75	0.18	129 ± 28.0	28 ± 1.0	4.6 ± 1.2
	100/1.00	0.24	156 ± 15.1	29 ± 1.2	5.4 ± 0.3
	100/1.25	0.30	173 ± 5.9	29 ± 4.8	6.2 ± 1.2
PA 10.10/ADR 4468	100/0.25	0.09	185 ± 27.3	27 ± 0.2	6.8 ± 1.1
	100/0.50	0.19	181 ± 28.1	29 ± 0.1	6.2 ± 1.0
	100/0.75	0.28	148 ± 14.1	28 ± 3.0	5.3 ± 0.1
	100/1.00	0.38	127 ± 38.8	25 ± 2.0	5.0 ± 1.2
	100/1.25	-	-	-	-

Φ: equivalent ratio (ratio of functional groups between chain-extender and polyamide 10.10); $\bar{M_{w}}$: weight average molar mass; $\bar{M_{n}}$: number average molar mass; D: polydispersity index; ^1^ SEC values (refractive index) are the mean of two measurements.

### 3.3. Oscillation Rheology (Plate–Plate)

**Figure 4:**
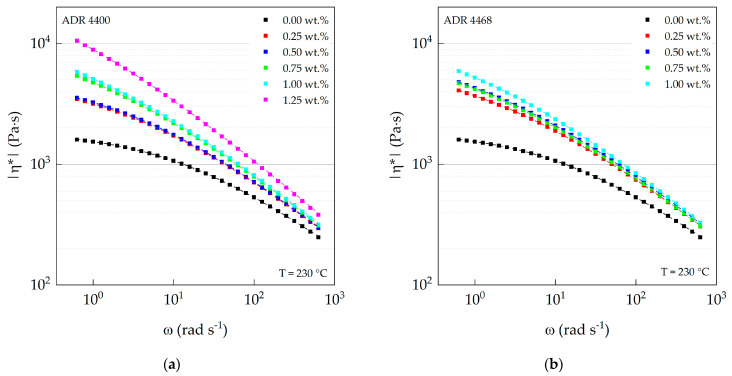

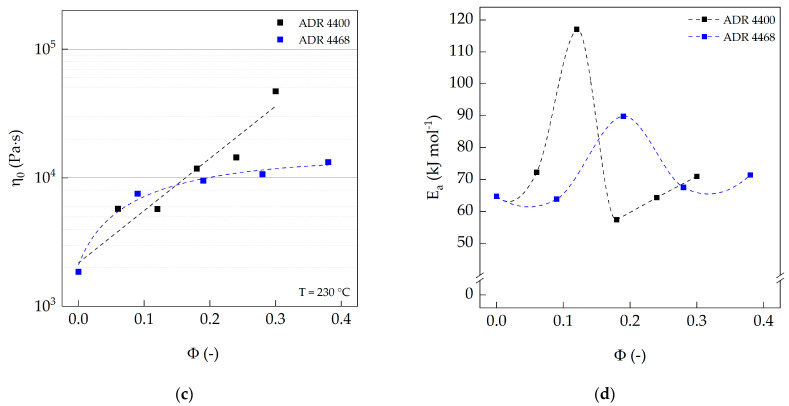
(**a**) Complex viscosity of ADR 4400–modified and (**b**) ADR 4468–modified PA 10.10 as a function of angular frequency; (**c**) zero shear viscosity of ADR 4400– and ADR 4468– modified PA 10.10 as a function of the equivalent ratio; (**d**) apparent activation energy $E_{a}$ of the modified PA 10.10 compounds as a function of the equivalent ratio.

The viscoelastic behavior of the different modified PA 10.10 compounds was analyzed by oscillation measurements. Figure 4a,b show the magnitude of the complex viscosity as a function of angular frequency of the two Joncryl^®^ types ADR 4400 and ADR 4468. The magnitude of the complex viscosity increased with increasing chain-extender content, especially in the low-frequency or shear range (first Newtonian plateau region). This is in good agreement with the average molar mass (Table 5) of the ADR 4400 compounds. At first glance, however, the data of the complex viscosity of the ADR 4468 type contradict the results measured with SEC, which show a maximum of the average molar mass at 0.25 wt.% ADR 4468 content. In comparison, the polymer chains are in the molten state and are not diluted, as required for SEC analyses. As mentioned above, Joncryl^®^ ADR 4468 contains more functional groups than ADR 4400, so the PA 10.10 compounds with ADR 4468 are shorter than those of ADR 4400 due to shear-induced degradation but still have a highly branched structure, which also leads to an increase in viscosity. This behavior was also observed with Joncryl^®^-modified PBT [59]. In addition, a more branched structure leads to more entanglement and also increases the viscosity in the lower frequency range [60].

The zero shear viscosities of modified polyamide 10.10 correlate with those of the melt flow rate, which was also measured but under different conditions. The viscosities increased with increasing amounts of chain-extender (Table 6). The polyamide modified with ADR 4400 showed higher values than PA 10.10 modified with ADR 4468, which can be attributed to the previously described shear-induced degradation during processing.

At higher frequencies, the viscosity of the compounds approximates with ADR 4400 and ADR 4468 due to the shear thinning behavior. The Carreau–Yasuda model was used to extrapolate the zero shear viscosities. The zero shear viscosity of the compounds with ADR 4400 as chain-extender increased linearly with increasing equivalent ratio of the functional groups (Figure 4c). In contrast, the compounds with ADR 4468 show no linear correlation between zero shear viscosity and the corresponding equivalent ratios. This can be explained by the competing processes between chain degradation (thermo-mechanical) [58] and increasing branching level and length of the branches (chemical reaction) during extrusion. The average molar masses of the ADR 4468 compounds decline linearly (Table 5) with increasing amounts of chain-extender, but we assume that they must exhibit a highly branched structure. The viscosities of the ADR 4468 compounds are lower compared to the ADR 4400 grades due to the constant decrease in average molar mass, but this can be compensated by entanglements caused by the increased degree of branching of the ADR 4468 types.

The apparent activation energies $E_{a}$ of the epoxy-modified PA 10.10 are shown in Figure 4d. The determined apparent activation energy of PA 10.10 (processed), 64.7 kJ mol^−1^, is higher than the value of 50.5 kJ mol^−1^ measured by Nishitani et al. [61] and also that of PA 6.10, 57 kJ mol^−1^ [62]. The apparent activation energies of ADR 4400 and ADR 4468 show a maximum at an equivalence ratio of 0.12 (0.5 wt.% ADR 4400) and 0.19 (0.5 wt.% ADR 4468). It was found that for different polyolefin types, the apparent flow activation energy increases with long chain branching (LCB) [63,64]. Munari et al. [65,66,67] determined the apparent activation energies for different linear and branched polyester types. Depending on the polyester studied, different conclusions can be drawn [65,66,67]. For branched poly (buthylene therephtalate) (PBT), an increase in $E_{a}$ was reported compared to the linear types [65]. Only a small increase was observed for PET [66], while the activation energy did not change for branched poly (buthylene isophthalate) (PBI) [67]. They conclude that the activation energy is independent of the molar mass and depends only on local motion of the chain.

For the modification of PLA and polybutylene (terephthalate) (PBAT), Al-Itry et al. [68] used Joncryl^®^ ADR 4368, which has the same functionality as the Joncryl^®^ used in this study and differs only in molar mass. Compared to our studies, the apparent activation energy of PLA and PBAT increased with Joncryl^®^ content and branching degree, respectively [68]. For a better and deeper understanding of the local chain motions and the branching modes involved, further analyses need to be performed.

**Figure 5:**
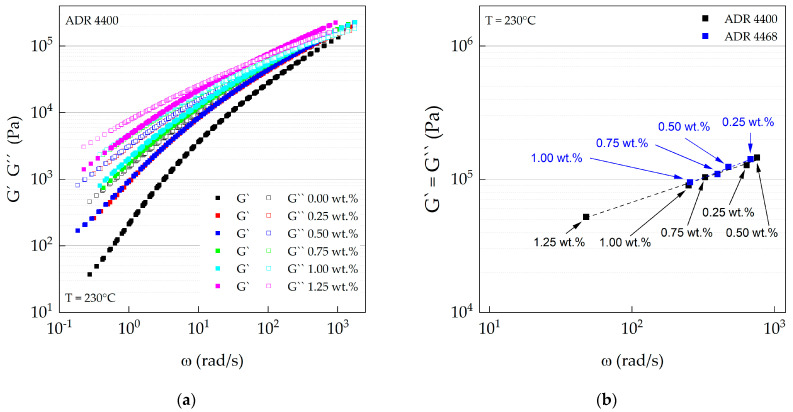
(**a**) Master curves of ADR 4400–modified PA 10.10 as a function of angular frequency; (**b**) cross-over points (G′ = G″) of ADR 4400– and ADR 4468–modified PA 10.10 as a function of angular frequency.

To verify whether chain-extension occurred for both Joncryl^®^ types, the cross-over points of the different compounds were determined by establishing master curves at 230 °C (Figure 5a,b). With an increasing amount of chain-extender, the cross-over point shifts towards lower angular frequencies and moduli. This confirms that as the content of chain-extenders increases, the average molar masses increase and their distribution (D) is broadened. The results from rheological measurements are in a good agreement with the data from GPC measurements for the ADR 4400-modified PA 10.10 shown before. Kamleitner et al. [69] came to the same conclusion for long chain branched (LCB) isotactic polypropylene (iPP).

### 3.4. Attenuated Total Reflectance (ATR)

ATR spectroscopy was used to quantitatively analyze whether chain-extension, e.g., branching of polyamide 10.10, occurred during compounding. The same samples (thin films) were used for ATR measurement as well as rheological measurement. The preparation of all samples was the same as described in Section 2.2.2.

Due to the same thermal process and especially cooling time (cooling to ambient temperature), no differences between the two crystal forms, pseudo γ-hexagonal and triclinic α-crystal form, were observed for the samples [47]. All intensities and wavenumbers corresponding to their specific crystal form were nearly identical for each compound, as described previously.

**Figure 6:**
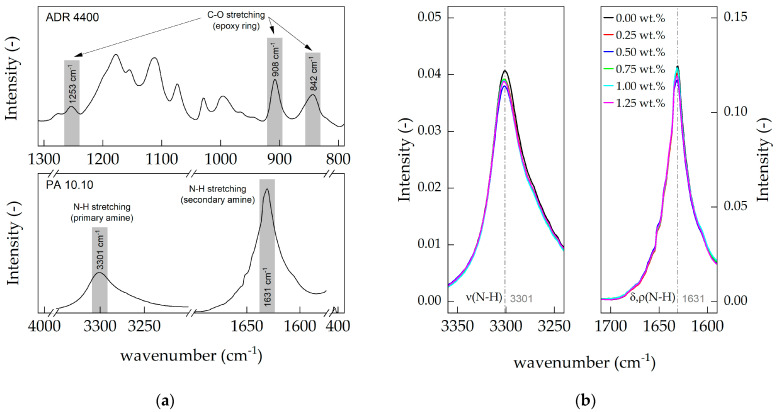
(**a**) FTIR/ATR spectra of Joncryl^®^ ADR 4400 and PA 10.10 at representative peak positions; (**b**) ATR spectra of the ADR 4400-modified compounds in the primary amine (3350–3250 cm^−1^) and secondary amine (1700–1600 cm^−1^) region of polyamide 10.10.

No detectable changes were observed in the compounds at 1255, 910 and 840 cm^−1^ of C-O stretching in the epoxy ring (Figure 6a). This means that the concentration of Joncryl^®^ was too low to be detected or that all epoxy groups of Joncryl^®^ were consumed during the compounding process, as shown by Costa et al. [57]. As the amount of Joncryl^®^ in polyamide 10.10 increased, the N-H stretching (3301 cm^−1^) of the primary amine bond decreased (Figure 6b). Zhang et al. [70] came to the same conclusion for polyamide 6. Moreover, the intensity of N-H stretching of the secondary amine at wavenumber 1630 cm^−1^ also decreased with increasing chain-extender content. Both results indicate that chain-extension (primary amine at 3301 cm^−1^) and branching (secondary amine at 1600 cm^−1^) occur simultaneously in polyamide 10.10.

## 4. Conclusions

It was shown that polyamide 10.10 can be modified in its thermal and rheological properties by adding two chain-extenders (Joncryl^®^ ADR 4400 and ADR 4468). With increasing chain-extender content, the weight average molar mass of the ADR 4400 polyamide 10.10 compounds can be increased, whereas it decreases with the ADR 4468 type. The high degree of branching assumed for both ADR types leads to an increase in viscosity and melt strength.

With increasing chain-extender content, the degree of branching can be increased, while the total crystallinity decreases and the melting temperature remains constant. During the second heating run (Appendix A), a bimodal melting peak consisting of the pseudo γ-hexagonal and the triclinic α-crystal was formed due to the high degree of branching. The cumulative enthalpy of fusion in the second heating run remained constant, while the γ-crystal form (metastable) increased and the α-crystal form decreased.

The viscosity of the modified polyamide 10.10 can be increased by the addition of chain-extenders up to a concentration of 1.25 wt.%. In the lower Joncryl^®^ concentration range, ADR 4468 shows higher viscosity values due to the higher number average functionality of 9 compared to 5 of the ADR 4400 type. At a concentration of 0.75 wt.% this behavior is reversed, due to the competing thermal degradation and increasing degree of branching of the ADR 4468 type (during processing). Therefore, the Joncryl^®^ ADR 4400 compounds show a better overall performance. The flow activation energy for both Joncryl^®^-modified compounds shows a maximum at 0.50 wt.% (Φ = 0.12 ADR 4400 and Φ = 0.19 ADR 4468).

In the ATR spectra, the C-O stretching of the epoxy ring was not visible, leading to the assumption that the concentration of the chain-extender was too low, or the epoxy was completely consumed during processing. The decrease in intensity of the primary and secondary amines indicates that chain-extension, e.g., branching, has taken place during processing. It can be concluded that modification with Joncryl^®^ leads to polyamides with a highly branched structure.

To determine the degree of branching and the length of the branches in detail, additional measurements must be carried out. Furthermore, the filaments produced from the compounds have to be tested for their mechanical properties, which are crucial for self-reinforced plastics.

## Figures and Tables

**Table 1:** Overall properties of the two Joncryl^®^ chain-extender types [31,32].

Chain-Extender	$M_{w}$	ρ ^1^	EEW	$f_{n}^{CE}$ ^2^	Non Vol by GC	$T_{g}$	$T_{m}$
	[g mol^−1^]	[g cm^−3^]	[g mol^−1^]	[-]	[wt.%]	[°C]	[°C]
Joncryl^®^ ADR 4400	7100	1.16	485	5	>99	65	>100
Joncryl^®^ ADR 4468	7250	1.16	310	9	>99	59	-

$M_{w}$: Molar mass; ρ: density; EEW: epoxy equivalent weight; $f_{n}^{CE}$: number average functional groups; Non Vol by GC: non-volatile organic compound measured with gas chromatography; $T_{g}$: glass transition temperature; $T_{m}$: melting temperature; ^1^ property measured at 20 °C; ^2^ quantity of the number average functional groups taken from the source [33]; all data, except the functionalities, are taken from the product specification sheet (PS), technical information sheet (TI) or the product safety data sheet (SDS) provided by the distributor or manufacturer.

**Table 2:** Temperature profile of the intermeshing twin-screw extruder with its characteristic primary functions in terms of screw design.

Zone	Die	9	8	7	6	5	4	3	2	1	Feeder
Temperature (°C)	265	265	260	260	260	260	260	250	240	230	220
Screw design (-)	Conveying		Mixing	Conveying			Mixing and dispersion	Kneading		Conveying	

**Table 3:** FTIR data of polyamide 10.10 [3,36] and Joncryl^®^ ADR types.

Reference Band	PA 10.10	Band Assignment	Joncryl^®^ ADR 4400 and 4468 Type	Band Assignment
[cm^−1^]	[cm^−1^]	[-]	[cm^−1^]	[-]
General				
3300 [37]	3303	N-H stretch hydrogen bonded	1597 [38]	C-C stretching in phenyl
3070 [37]	3072	N-H stretch and amide II overtone	1489 [38]	C-C stretching in phenyl
2935 [37]	2920	CH_2_ asymmetric stretching	1447 [38]	CH_3_ scissoring vibration
2860 [37]	2851	CH_2_ symmetric stretching	1250 [38,39]	Stretching in epoxy C-O
1741 [40]	1741	C=O stretch (ester)	905 [38]	Stretching in epoxy C-O
1660 [37]	1637	Amide I: C=O stretch	844 [38]	Stretching in epoxy C-O
1530 [37]	1535	Amide II: N-H in plane bending coupled with C-N and C-O stretch		
1170 [41]	1165	CO-NH skeletal, crystalline		
1123 [41]	1122	Amorphous		
α-structure				
1466 [42]	1466	CH_2_ scissoring not adjacent to the amide group		
1416 [43]	1419 (mi)	CH_2_ scissoring		
1373 [43]	1372	Amide III and CH_2_ wagging		
1262 [37]	-	Amide III		
1200 [44]	1191	CH_2_ twist-wagging		
959 [44]	959 (vw)	CO-NH in plane (shoulder)		
936 [41]	936	Vibration of the N-vicinal CH_2_ group coupled amide III “crystal band”		
γ-structure				
1439 [45]	1437	CH_2_ scissor vibration		
1369 [43]	1360	CH_2_ twist-wagging		
1329 [41]	-	C-H deformation		
1255 [42]	-	Skeletal C-C stretch		
1236 [46]	1237	CH_2_ twist-wagging		
976 [44]	-	CO-NH in-plane		
α- and γ-structure				
730 [37]	721	Rocking mode of CH_2_		

(vw): very weak; (mi): middle.

**Table 4:** DSC results of the different chain-extended PA 10.10 compounds (1. heating run with a heating rate of 20 K min^−1^).

Compound	PA 10.10/ADR Ratio	$T_{pc}$	${\Delta H}_{cc}$	$T_{{pm}^{2}}^{\left(\alpha \right)}$	${\Delta H}_{m}^{\left(\gamma +\alpha \right)}$	$X_{c}^{\left(total\right)}$
	[wt.%]	[°C]	[J g^−1^]	[°C]	[J g^−1^]	[%]
PA 10.10 (processed)	100	-	-	206	71.47	29.29
PA 10.10/ADR 4400	100/0.25	172	−1.01	207	64.20	25.96
	100/0.50	168	−0.70	207	67.58	27.69
	100/0.75	179	−0.73	206	57.01	23.24
	100/1.00	181	−0.56	205	52.63	21.55
	100/1.25	172	−0.10	208	52.73	21.86
Joncryl^®^ ADR 4400	100	-	-	152	3.63	-
PA 10.10/ADR 4468	100/0.25	177	−1.20	209	59.90	24.12
	100/0.50	173	−1.07	207	58.56	23.68
	100/0.75	176	−0.94	208	61.29	24.92
	100/1.00	175	−0.87	208	59.20	24.13
	100/1.25	-	-	-	-	-
Joncryl^®^ ADR 4468	100	-	-	135	2.34	-

$T_{pc}$: crystallization peak temperature; ${∆H}_{cc}$: enthalpy change during the cold crystallization process; $T_{{pm}^{2}}^{\left(\alpha \right)}$: second melting peak temperature; ${∆H}_{m}^{\left(\gamma +\alpha \right)}$: enthalpy change (γ+α-crystal form) during the melting process; $X_{c}^{\left(total\right)}$: overall crystallinity (γ+α-crystal form + cold crystallization if present).

**Table 6:** Viscoelastic properties of the modified polyamide 10.10 compounds (230 °C).

Compound	PA 10.10/ADR Ratio	$\eta_{0}$	λ	n	a	$E_{a}$	$G^{\prime }=G^{\prime \prime }$	MFR^1^	$\eta^{MFR}$
	[wt.%]	[Pa s]	[-]	[-]	[-]	[kJ mol^−1^]	[Pa]	[g 10 min^−1^]	[Pa s]
PA 10.10 (processed)	100	1857	0.092	0.528	0.572	64.7	-	9.8 ± 0.2	974
PA 10.10/ADR 4400	100/0.25	5738	0.259	0.445	0.420	72.3	127,508	3.1 ± 0.2	2896
	100/0.50	5717	0.336	0.471	0.449	117.1	145,438	2.0 ± 0.1	4611
	100/0.75	11,799	1.010	0.452	0.420	57.4	103,780	1.3 ± 0.1	7297
	100/1.00	14,420	1.546	0.461	0.407	64.3	90,019	0.6 ± 0.1	15,509
	100/1.25	47,062	4.761	0.414	0.355	71.0	52,177	0.5 ± 0.0	19,653
PA 10.10/ADR 4468	100/0.25	7520	0.470	0.461	0.415	63.9	141,612	2.0 ± 0.4	4302
	100/0.50	9505	0.661	0.456	0.417	89.8	123,839	1.4 ± 0.1	6459
	100/0.75	10,683	0.510	0.413	0.362	67.6	109,398	1.4 ± 0.1	6646
	100/1.00	13,245	1.121	0.451	0.423	71.4	95,137	1.1 ± 0.1	8220
	100/1.25	-	-	-	-	-	-	-	-

$\eta_{0}$: zero shear viscosity, λ: time constant, n: flow index (0 < n < 1); a: dimensionless factor (transition between the first Newtonian plateau and the shear thinning behavior); (G′ = G″): cross-over point of the modified PA 10.10; MFR : melt flow rate; $\eta^{MFR}$ : viscosity assessed under MFR conditions; ^1^ compounds are pre-dried in an oven for 4 h/80 °C; measurements are performed at 235 °C/2.16 kg; pre-heating time 300 s; die geometry d = 2.095 mm with a length of 8 mm; 10 mm cutting length.

## Data Availability

The data presented in this study are available on request.

## Appendix A

**Table A1:** DSC results of the different chain-extended polyamide 10.10 compounds (2. heating run; heating rate 20 K min^−1^).

Compound	PA 10.10/ADR Ratio	$T_{im}$	$T_{{pm}^{1}}^{\left(\gamma \right)}$	$T_{{pm}^{2}}^{\left(\alpha \right)}$	$T_{fm}$	${\Delta H}_{m}^{\left(\gamma +\alpha \right)}$	$X_{c}^{\left(total\right)}$
	[wt.%]	[°C]	[°C]	[°C]	[°C]	[J g^−1^]	[%]
PA 10.10 (processed)	100	179	191	201	207	81.36	33.3
PA 10.10/ADR 4400	100/0.25	180	193	202	211	80.09	32.8
	100/0.50	182	193	202	210	78.81	32.3
	100/0.75	179	193	202	211	80.66	33.1
	100/1.00	180	193	200	209	82.82	33.9
	100/1.25	181	194	200	209	78.48	32.2
Joncryl^®^ ADR 4400	100	-	-	-	-	-	-
PA 10.10/ADR 4468	100/0.25	183	193	202	211	73.73	30.2
	100/0.50	183	193	201	209	77.01	31.6
	100/0.75	181	193	201	211	81.19	33.3
	100/1.00	181	193	201	209	80.16	32.9
	100/1.25	-	-	-	-	-	-
Joncryl^®^ ADR 4468	100	-	-	-	-	-	-

$T_{im}$: onset melting temperature; $T_{{pm}^{1}}^{\left(\gamma \right)}$: first melting peak temperature; $T_{{pm}^{2}}^{\left(\alpha \right)}$: second melting peak temperature; $T_{fm}$: end temperature melting; ${∆H}_{m}^{\left(\gamma +\alpha \right)}$: enthalpy change (γ+α-crystal form) during the melting process; $X_{c}^{\left(total\right)}$: overall crystallinity (γ+α-crystal form).

**Table A2:** Apparent flow activation energy of the different modified PA 10.10 compounds at the glass transition region (1. heating run).

Compound	PA 10.10/ADR Ratio	$E_{a}^{T_{g}\left(Moynihan\right)}$	$E_{a}^{T_{g}\left(Kissinger\right)}$
	[wt.%]	[kJ mol^−1^]	[kJ mol^−1^]
PA 10.10 (processed)	100	39.0 ± 7.4	33.2 ± 7.4
PA 10.10/ADR 4400	100/0.25	67.2 ± 9.6	61.6 ± 9.6
	100/0.50	73.6 ± 6.6	68.0 ± 6.7
	100/0.75	68.9 ± 3.2	63.3 ± 3.3
	100/1.00	62.1 ± 7.9	56.6 ± 7.9
	100/1.25	57.9 ± 5.0	52.3 ± 5.0
PA 10.10/ADR 4468	100/0.25	60.1 ± 7.8	54.5 ± 7.9
	100/0.50	70.1 ± 7.7	64.6 ± 7.7
	100/0.75	66.7 ± 6.6	61.1 ± 6.7
	100/1.00	61.6 ± 10.1	56.0 ± 10.1
	100/1.25	-	-

$E_{a}^{T_{g}\left(Moynihan\right)}$: apparent flow activation energy (Moynihan model); $E_{a}^{T_{g}\left(Kissinger\right)}$: apparent flow activation energy (Kissinger model).

**Table A3:** Process parameters of the Joncryl^®^-modified polyamide 10.10 compounds.

Compound	PA 10.10/ADR Ratio	Torque	Die Pressure
	[wt.%]	[Nm]	[bar]
PA 10.10 (processed)	100	34.3	11
PA 10.10/ADR 4400	100/0.25	35.0	14
	100/0.50	35.0	17
	100/0.75	35.7	22
	100/1.00	36.4	28
	100/1.25	37.8	30
PA 10.10/ADR 4468	100/0.25	37.8	14
	100/0.50	39.2	16
	100/0.75	37.1	11
	100/1.00	37.8	14
	100/1.25	-	-
